# Virus diversity and activity is driven by snowmelt and host dynamics in a high-altitude watershed soil ecosystem

**DOI:** 10.1186/s40168-023-01666-z

**Published:** 2023-10-27

**Authors:** Clement Coclet, Patrick O. Sorensen, Ulas Karaoz, Shi Wang, Eoin L. Brodie, Emiley A. Eloe-Fadrosh, Simon Roux

**Affiliations:** 1 DOE Joint Genome Institute, Lawrence Berkeley National Laboratory, Berkeley, CA USA; 2 Earth and Environmental Sciences Area, Lawrence Berkeley National Laboratory, Berkeley, CA USA; 3 Department of Environmental Science, Policy and Management, University of California, Berkeley, Berkeley, CA USA

**Keywords:** Phages, Viruses, Metagenomics and metatranscriptomics, Virus activity, Virus-host interactions, Mountainous watershed, Soils, Seasonal dynamics

## Abstract

**Background:**

Viruses impact nearly all organisms on Earth, including microbial communities and their associated biogeochemical processes. In soils, highly diverse viral communities have been identified, with a global distribution seemingly driven by multiple biotic and abiotic factors, especially soil temperature and moisture. However, our current understanding of the stability of soil viral communities across time and their response to strong seasonal changes in environmental parameters remains limited. Here, we investigated the diversity and activity of environmental soil DNA and RNA viruses, focusing especially on bacteriophages, across dynamics’ seasonal changes in a snow-dominated mountainous watershed by examining paired metagenomes and metatranscriptomes.

**Results:**

We identified a large number of DNA and RNA viruses taxonomically divergent from existing environmental viruses, including a significant proportion of fungal RNA viruses, and a large and unsuspected diversity of positive single-stranded RNA phages (*Leviviricetes*), highlighting the under-characterization of the global soil virosphere. Among these, we were able to distinguish subsets of active DNA and RNA phages that changed across seasons, consistent with a “seed-bank” viral community structure in which new phage activity, for example, replication and host lysis, is sequentially triggered by changes in environmental conditions. At the population level, we further identified virus-host dynamics matching two existing ecological models: “Kill-The-Winner” which proposes that lytic phages are actively infecting abundant bacteria, and “Piggyback-The-Persistent” which argues that when the host is growing slowly, it is more beneficial to remain in a dormant state. The former was associated with summer months of high and rapid microbial activity, and the latter with winter months of limited and slow host growth.

**Conclusion:**

Taken together, these results suggest that the high diversity of viruses in soils is likely associated with a broad range of host interaction types each adapted to specific host ecological strategies and environmental conditions. As our understanding of how environmental and host factors drive viral activity in soil ecosystems progresses, integrating these viral impacts in complex natural microbiome models will be key to accurately predict ecosystem biogeochemistry.

Video Abstract

**Supplementary Information:**

The online version contains supplementary material available at 10.1186/s40168-023-01666-z.

## Background

Soil microbiomes represent a major reservoir of microbial diversity on Earth and provide many critical ecosystem services such as driving the transformation of carbon and other nutrients and sustaining plant growth [1]. Soil ecosystems, found across a large range of environments including deserts, wetlands, forests, and mountains, are vulnerable to climate change [2–5]. Mountainous ecosystems in particular are impacted by unprecedented snowpack reductions and earlier spring snowmelt [3, 6, 7], which can trigger rapid collapse of the microbial biomass and abrupt changes in the composition and functioning of soil microbiomes [6]. However, predicting the impact of environmental changes on soil microbiomes remains challenging [2, 3, 5], and holistic studies, including soil virus-microbe interactions, are needed to elucidate the ecological consequences of climate change on these ecosystems.

Viruses are commonly found in all environments [8], from the human gut to the ocean, and in many different soil types including agricultural soils [9–11], grasslands [12–17], and deserts [18–20]. The complexity of habitats and the wide variety of cellular microorganisms found in soils, including protozoa, algae, fungi, bacteria, and archaea, create an environment that promotes high viral diversity [21]. These viruses may play essential roles in soil biogeochemistry and ecosystem functions associated with microbes [21–23]. Viruses infecting bacteria and archaea (hereafter referred to as phages) are the most common and diverse group of viruses identified in soil and can harbor various virion morphologies and genome types including double-stranded DNA (dsDNA), single-stranded DNA (ssDNA), single-stranded RNA (ssRNA), and double-stranded RNA (dsRNA) genomes [24]. Soil viruses are highly abundant and diverse; however, public databases still capture only a fraction of this diversity (mainly dsDNA viruses) [8, 25–27], due to a combination of biological biases and methodological limitations. Virus genome representation in public databases is also biased towards dsDNA phages, which represent the overwhelming majority of viruses reported in the soil to date [21, 23], while our current knowledge of the diversity and ecological role of soil RNA and ssDNA viruses remains limited [13, 14, 28–30].

Previous soil viral ecology studies based on viral particle counts and/or multi-omics analyses have suggested that soil warming, permafrost thaw, and shifts in soil moisture directly and/or indirectly influenced soil viral diversity [13, 31–35]. Overall, spatiotemporal patterns in soil viral community composition could be associated with abiotic (soil temperature and depth, pH, and moisture) [9, 13, 27, 29, 33, 36–40] and biotic factors such as host community composition [36, 41, 42]. Viruses may impact soil ecosystem functioning especially through viral infections of key biogeochemical-cycling microbes, and these viral-host dynamics may change with environmental conditions [36–38]. Additionally, growing evidence of viral populations carrying auxiliary metabolic genes, i.e., viral-encoded metabolic genes that could provide a fitness advantage to their hosts during infection, provide a complementary way by which viruses likely influence soil biogeochemical cycling [13, 33]. While studies of soil viral ecology have highlighted the potential influence of viruses on host community structure, nutrient cycling, and other ecosystem processes, major gaps remain in our understanding of soil viral ecology and diversity. In particular, the activity and dynamics of phages across seasons is poorly understood, especially in mountainous environments where abrupt seasonal changes occur. Additionally, the ecological models that describe phage-bacteria interactions, i.e., lytic predation according to classical predator–prey Kill-the-Winner (KTW) dynamics and temperate infection according to Piggyback-the-Winner (PTW) appear to be conflicting hypotheses as they both occur in same types of environments but can lead to opposite outcomes. New paradigms, such as the Piggyback-the-Persistent (PTP) strategy, which involves viruses lysogenically infecting hosts that are consistently present at low abundances [43], and Piggyback-the-Loser (PTL), where viral lysogeny in “loser” or dying hosts is unlikely due to a lack of benefits and inconsistencies with natural selection [44], offer alternative explanations that better align with field observations. These emerging models challenge the conventional understanding, as they coexist in similar environments and can lead to divergent outcomes. Moreover, the mechanisms that trigger the lytic-lysogenic switch remain mostly unknown [45, 46]. Thus, the direct and indirect impacts of climate change on mountainous soil viruses, and the subsequent repercussions on soil microbiome and metabolic processes, are currently unknown.

Here, we aimed to explore how strong seasonal disturbance, in this case snowmelt in a mountainous watershed, influences the diversity, composition, and activity of soil viruses. To this end, we leveraged existing metagenomic and metranscriptomic data from soil samples collected at the East River Watershed (ERW) in Colorado (USA) as part of a coordinated and multiscale research that integrates hydrological, biogeochemical, and microbiological studies [47]. Previous investigations demonstrated the substantial impact of seasonal snowmelt on the diversity and activity of resident bacterial, archaeal, and fungi communities [6]. Consequently, we anticipated that an integrated study across metagenomes and metatranscriptomes would reveal a broad diversity of DNA and RNA viruses infecting both prokaryotic and eukaryotic hosts and that viral communities in ERW would substantially change throughout the seasons, i.e., before and after snowmelt. We also hypothesized that these complex soil communities would likely include a mix of viruses with different infection strategies (e.g., KTW, PTW, PTP, or PTL, see above) that could be resolved by analyzing the abundance and activity of individual viruses or viral groups across time. These data and analyses thus contribute to our collective understanding of the global soil virosphere, provide a more comprehensive understanding on how virus-host interactions evolve throughout seasons, and highlight the potential role and impact of viruses on soil ecological processes, particularly their influence on microbial communities and soil biogeochemical cycling.

## Methods

### Field site description

The East River Watershed (ERW) is located in Gunnison County, Colorado, near the town of Crested Butte (38°57.5’ N, 106°59.3’ W) and is the location of the Rocky Mountain Biological Laboratory. Elevations within the ERW range from 2750 to 4000 m. Snow cover in winter typically persists 4 to 6 months (e.g., November through May) followed by an arid summer with intermittent, monsoonal rain events that occur from July through September. The minimum annual daily air temperature occurs in January (− 14 ± 3° C), whereas the maximum daily air temperature typically occurs in July (23.5 ± 3° C). Plant composition at this location is a mixed, montane meadow community comprised of perennial bunchgrasses (e.g., *Festuca arizonica*), forbs (e.g., *Lupinus* spp., *Potentilla gracilis*,* Veratrum californicum*), and shrubs (*Artemesia tridentata*).

### Soil sampling

**Fig. 1:**
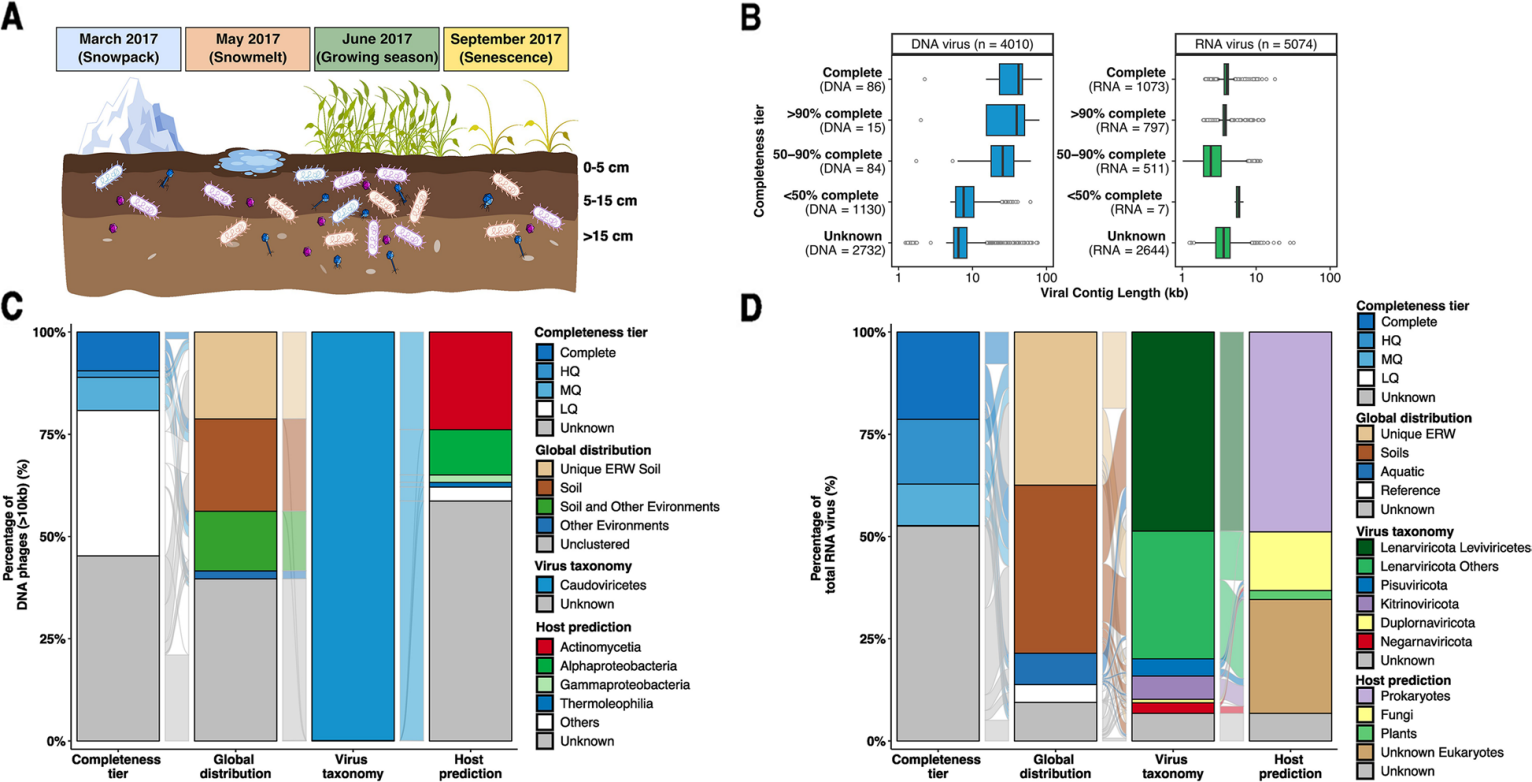
Overview of sampling strategy and general features of East River Watershed (ERW) DNA and RNA viruses. **A** Visual schematic of the sampling strategy. Bulk soil samples were collected in the ERW, Colorado on four dates, starting first at peak winter snow depth (March 7, 2017), during the snowmelt period (May 9, 2017), following the complete loss of snow and the start of the plant growing season (June 9, 2017), and finishing in autumn after plant senescence (September 15, 2017), at three discrete depth increments; 0 to 5 cm, 5 to 15 cm, and 15 cm + below the soil surface (Supplementary Tables 1 and 2). During snow-free times of the year (i.e., September and June), soils were collected at the upslope and downslope while during periods of winter snow cover (i.e., March and May), snow-pits were dug down to the soil surface, eventually yielding 46 and 43 metagenomes and metatranscriptomes, respectively. **B** Length distribution of the different quality tiers of DNA (blue) and RNA (green) vOTUs, based on their estimated completeness assessed by CheckV. HQ high-quality (≥ 90% complete), MQ medium-quality (≥ 50% complete), and LQ low-quality (< 50% complete). The LQ RNA vOTUs appear to be uniquely associated with larger RNA virus genomes (Supplementary Table 4). **C** Completeness, global distribution, virus taxonomy, and host taxonomy for DNA vOTUs ≥ 10kbp. Global distribution is based on shared clusters from a vContact2 analysis using only DNA vOTUs ≥ 10kbp, and virus and host taxonomy are based on GeNomad and iPHoP, respectively (see the “Methods” section). Completeness, global distribution, virus taxonomy, and host taxonomy for all DNA vOTUs (including ones < 10kbp) are presented in Supplementary Fig. 4. The spaces between the different branches represent the transition (or connection) of each vOTU between categories.** D** Completeness, global distribution, virus taxonomy, and host taxonomy for RNA vOTUs. Global distribution is assessed by genome-based clustering, and virus and host taxonomy are based on GeNomad and iPHoP and refined using phylogenetic analyses of the RNA virus marker gene RdRP (RNA-dependent RNA polymerase, see the “Methods” section)

Soils were sampled from upland hillslope-to-riparian floodplain transect that was adjacent to the main stem of the East River (elevation ~ 2775 m). Soil samples were collected using a 4-cm diameter soil bulk density corer on four dates starting at maximum winter snow depth (March 7, 2017), during the peak snowmelt period (May 9, 2017), during the plant growing season after the complete loss of snowpack (June 9, 2017), and lastly in autumn after plant senescence (September 16, 2017) (Fig. 1A, Supplementary Fig. 1A and Supplementary Tables 1 and 2). When there was no snowpack (June and September), soil samples were collected from 6 locations separated by approximately 10 m at the upslope and downslope while during periods of winter snow cover (approximately 1.5 m of snowpack, March and May), and snow-pits were dug down to the soil surface at locations adjacent to previous sampling sites in order to sample soils from beneath the snowpack. The 48 soil samples were split into three discrete depth increments; 0 to 5 cm, 5 to 15 cm, and 15 cm + below the soil surface. A ~ 10 g subsample from each soil core at each depth was frozen immediately on dry ice in the field and archived at − 80 °C for metagenome and metatranscriptome analyses. From these samples, a total of 46 and 43 paired metagenomes and metatranscriptomes, respectively, were leveraged for this current study, including 7 from March, 9 from September (6 metatranscriptomes only), and 15 each for May and June. More information on individual samples including specific location, plot number, depth, and accession numbers across databases is available in Supplementary Tables 1 and 2, with extended physicochemical parameters available on ESS-DIVE (https://ess-dive.lbl.gov).

### Nucleic acid extraction

Nucleic acids were extracted on ice from 5 to 7 technical replicates of each soil sample by adding 0.5 mL phenol:chloroform:isoamyl alcohol (25:24:1) (Sigma-Aldrich, St. Louis, MO, USA) to 0.5 g of soil in a 2 ml Lysing Matrix E tube (MP Biomedicals, Solon, OH, USA), followed by addition of 0.5 mL of CTAB buffer (5% CTAB, 0.25 M phosphate buffer pH 8.0, 0.3 M NaCl) and 50 μL of 0.1 M aluminum ammonium sulfate. The samples were homogenized at 5.5 m/s for 30 s in a FastPrep-24 instrument (MP Biomedicals, Solon, OH, USA), then centrifuged at 16 K g for 5 min at 4℃. The aqueous phase was removed and transferred to MaxTract High Density 2 mL tubes (Qiagen Inc, Valencia, CA, USA). Samples were then extracted a second time as described above, and the aqueous phase from the repeated extractions for each sample was combined. Sodium acetate (3 M sodium acetate, 1/10th volume of total aqueous phase) and ice-cold ethanol (100%, 2X volume of total aqueous phase) were added, the samples were vortexed briefly, and a crude nucleic acid pellet was precipitated overnight at − 20 °C. Following overnight precipitation, technical replicates for each soil sample were combined, then the separation of DNA and RNA was completed using the AllPrep DNA/RNA Mini Kit (Qiagen Inc., Valencia, CA, USA). The amount of DNA or RNA extracted was quantified using the Qubit 1X dsDNA Broad Range Kit or Qubit RNA High Sensitivity Kit, respectively (ThermoFisher Scientific). DNA and RNA quality were assessed using a 2100 Bioanalyzer instrument (Agilent Technologies, Santa Clara, USA). DNA and RNA were stored at − 80 °C prior to metagenome and metatranscriptome analyses (described below).

### Library preparation and sequencing

Sequence data was generated at the DOE Joint Genome Institute (JGI) using Illumina technology. For metagenomes, library preparation for Illumina sequencing was performed using the Kapa Biosystems library preparation kit. Briefly, DNA was sheared using a Covaris LE220 focused-ultrasonicator. The sheared DNA fragments were size selected by double-SPRI and then the selected fragments were end-repaired, A-tailed, and ligated with Illumina compatible sequencing adaptors from IDT containing a unique molecular index barcode for each sample library. The prepared libraries were quantified using KAPA Biosystems’ next-generation sequencing library qPCR kit and run on a Roche LightCycler 480 real-time PCR instrument. Sequencing of the flowcell was performed on the Illumina NovaSeq sequencer using NovaSeq XP V1 reagent kits, S4 flowcell, following a 2 × 151 indexed run recipe. For metatranscriptomes, rRNA was depleted using Illumina’s Ribo-Zero rRNA Removal Kit (Bacteria), and stranded cDNA libraries were generated using the Illumina TruSeq Stranded Total RNA kit. Briefly, the rRNA-depleted RNA was fragmented and reversed transcribed using random hexamers and SSII (Invitrogen) followed by second-strand synthesis. The fragmented cDNA was treated with end-pair, A-tailing, adapter ligation, and 10 cycles of PCR. qPCR was used to determine the concentration of the libraries. Sequencing of the flowcell was performed on the Illumina NovaSeq sequencer using NovaSeq XP V1 reagent kits, S4 flowcell, following a 2 × 151 indexed run recipe.

### Assembly and annotation of metagenomes and metatranscriptomes

Metagenome libraries were filtered and assembled using the DOE JGI Metagenome Workflow [48]. Briefly, BBDuk (version 38, https://jgi.doe.gov/data-and-tools/bbtools/) was used to remove contaminants via k-mer matching, trim reads that contained adapter sequence, right quality trim reads where quality drops to 0, and remove reads that contained 4 or more “N” bases or had an average quality score across the read less than 3 or had a minimum length <  = 51 bp or 33% of the full read length. Reads aligned with BBMap (https://jgi.doe.gov/data-and-tools/bbtools/) to masked human, cat, dog, and mouse references at 93% identity or to common microbial contaminants were excluded from downstream analyses. Specific commands and options used for read filtering are available for each library on the JGI Data portal (https://data.jgi.doe.gov), under proposal ID 503568 (https://genome.jgi.doe.gov/portal/Thesynhydregimes/Thesynhydregimes.info.html). Filtered reads were error-corrected using bfc (version r181) with bfc -1 -s 10 g -k 21 -t 10 [49] and then assembled with metaSPAdes [50, 51] version 3.13.0 using the “metagenome” flag, running the assembly module only (i.e., without error correction) with kmer sizes of 33, 55, 77, 99, and 127. All contigs larger or equal to 200 bp were then annotated using the IMG pipeline v.4.16.4 [52]. Briefly, protein-coding genes were predicted with Prodigal v2.6.3 [53] and prokaryotic GeneMark.hmm v2.8 [54] and compared to COG 2003 [55], Pfam v30 [56], and IMG-NR 20180530 [48] using HMMER 3.1b2 [57] and lastal 914 [58] for taxonomic and functional annotation. Finally, predicted protein-coding genes were also assigned to KEGG Orthology (KO) terms [59] and Enzyme Commission (EC) numbers based on similarity to reference sequences in the IMG-NR 20180530 database [48].

In addition to these individual assemblies of metagenome libraries, a combined assembly of the metagenome libraries was performed using MetaHipmer, an assembly tool uniquely able to perform large combined metagenome assemblies [60]. Filtered reads from the libraries were pooled and used as input for metagenome assembly with MetaHipmer v1, using default parameters. Contigs larger or equal to 500 bp were then submitted to IMG for a similar functional and taxonomic annotation as previously described, using the IMG annotation pipeline v.5.0.23 [48, 61].

Metatranscriptome libraries were similarly processed using the default JGI workflow. BBDuk (version 38, https://jgi.doe.gov/data-and-tools/bbtools/) was used to remove contaminants and ribosomal RNA via k-mer matching, trim reads that contained adapter sequence, right quality trim reads where quality drops to 0, and remove reads that contained 1 or more “N” bases, had an average quality score across the read less than 10 or had a minimum length <  = 51 bp or 33% of the full read length, or were aligned by BBMap (https://jgi.doe.gov/data-and-tools/bbtools/) to masked human, cat, dog, and mouse references at 93% identity, to common microbial contaminants, or to ribosomal RNA sequences. Filtered reads were then de novo assembled with MEGAHIT v1.1.2 [62], using k-mer sizes of 23, 43, 63, 83, 103, and 123. Contigs larger or equal to 200 bp were then submitted to IMG for annotation as previously described, using the IMG annotation pipeline v4.16.5 [52].

### Viral sequence detection and vOTU-specific annotation

All contigs larger than 1 kbp from the individual assemblies of metagenomes and metatranscriptomes and from the combined assembly of metagenomes were processed with VirSorter2 (v2.0.beta) for virus sequence detection [63] (Supplementary Fig. 1B). These predictions were further refined by identifying and removing potential host contaminants using CheckV v0.8.1 [64], inspecting all predicted proviruses, i.e., cases in which a contig is predicted to harbor both host and viral region(s) to refine provirus boundaries, and removing if necessary all predicted viral sequences with similarity to Type VI Secretion Systems. CheckV v0.8.1 [64] was then used to estimate the completeness of all filtered predicted viral sequences, and only sequences larger or equal to 5 kbp or estimated to be at least 50% complete (AAI-based completeness) were retained. For metatranscriptomes, contigs detected as likely RNA viruses based on a custom identification of RdRP genes [28] were added to the filtered dataset obtained from the VirSorter 2 analysis.

The full dataset of predicted viral sequences was clustered into vOTUs following standard guidelines [65] at 95% ANI and 85% AF using Mummer 4.0.0b2 [66], and the longest sequence was selected as the representative for each vOTU. To complement the IMG functional annotation (see above), protein-coding genes from selected representatives were compared to proteins from RefSeq Virus r2016 [67] using Diamond 0.9.24 [68] (minimum score of 50), to the VOGdb v205 (https://vogdb.org) using HMMER 3.3.2 [57] (minimum score of 30), and to the Pdb70 v190918 [69], Pfam v32 [70], and SCOPe70 v1.75 [71] databases (database package downloaded in Feb. 2019 from the HH-Suite website) using Hhblits 3.1.0 [72] (minimum probability of 90), and to the PHROGS v4 database using MMseqs2 v14 [73], and an *e*-value cutoff of 1E − 6). For each predicted gene, a consensus functional annotation was obtained by considering hits obtained across all databases. Selected vOTU representatives were then further refined to identify and remove sequences only encoding putative Insertion Sequences based on annotation keywords “Transposase,” “insertion sequence,” and “insertion element,” as well as sequences without any annotated gene (i.e., only composed of predicted cds without any significant hit to any database). This led to a final dataset of 9321 vOTUs.

### Estimation of the relative abundances of vOTUs

To evaluate vOTU and individual gene coverage, reads from individual metagenome and metatranscriptome libraries were mapped to the vOTU representative sequences with BBMap v38 (default parameters, https://jgi.doe.gov/data-and-tools/bbtools/) (Supplementary Fig. 1B). For metatranscriptomes, samtools v1.13 [74] and bedtools v2.30.0 [75] were used to calculate strand-specific coverage for both full sequences and individual genes. All reads mapped to vOTUs were used to calculate the RPKM (reads per kilobase per million) value of each vOTU after normalizing by the sequence depth (per million reads) and the length of the contig (in kilobase). RPKM values were only considered if reads mapped on at least 10% of the contig length, and set at 0 otherwise.

#### Phylogenetic analyses, taxonomic assignment and host prediction

GeNomad (end-to-end) v1.0.0beta (https://github.com/apcamargo/genomad) was used for taxonomic classification (default parameters) of each vOTU [76]. iPHoP v0.9beta [77] was used to predict the host family of each DNA and RNA vOTU using a minimum score of 75, default parameters otherwise, and with the prediction with the highest score selected for each vOTU.

Phylogenetic analysis was performed using RdRP sequences and was used to complement and refine the taxonomic classification and the host prediction of RNA vOTUs. Previously published RdRP Hidden Markov Model (HMM) profile database [78, 79] were used to search for RdRP sequences using HMMER 3.3.2 [57] on predicted cds from ERW viral contigs, recovered as described above, and on predicted cds from contigs provided by Wolf, Starr, Hillary, and Callanan studies [14, 78–80]. We supplemented the data set with RdRP sequences collected from NCBI RefSeq Virus database r2016 [67] and group II intron reverse transcriptases (RT), used as an outgroup. Acceptance criteria for the RdRP profile searches were *E* value ≤ 1e − 12 and score ≥ 50. This analysis identified 28,916 non-redundant predicted RdRP proteins. The extracted RdRP sequences were clustered using MMseqs2 v14 [73] with a sequence similarity threshold of 0.5 and a coverage threshold of 80% to reduce the number of sequences to be aligned and included in the final trees. Representative RdRP sequences for each cluster were broadly assigned to the five major branches of RdRPs based on the best hit to the RdRP profiles [78] and aligned using MAFFT v7.505 [81] (default parameters). All alignments were trimmed using TrimAl v1.3 [82] with the -gappyout option, and used to reconstruct maximum likelihood trees using FastTree v2.3 [83], and rooted by RT sequences. Generated trees were visualized with *ggtree* package using R (CRAN) [84]. All branches with support values lower than 50 were collapsed with a custom *perl* script.

The procedure to associate each cluster representative to a taxon and predicted host across the trees relies on the tree topology (i.e., monophyletic clades) and leverages existing taxonomic and host prediction information from RefSeq Virus r2016. Briefly, all sequences belonging to a monophyletic clade in which all reference sequences are affiliated to a single taxon *T* or connected to a single host H were also assigned to the same taxon *T* or host H. A custom *perl* script was ran on each phylogenetic tree to apply this logic based on the reference taxonomy and the host information. All taxonomic and host prediction information was then propagated to all cluster members based on the annotation of the representative (i.e., the cluster member included in the tree).

### Ecological distribution analyses

For DNA phages, a network analysis using vContact v2.0 [85] with “ − rel-mode Diamond,” “ − vcs-mode ClusterONE,” and all other settings set to default was used to compare the ≥ 10kbp DNA ERW virus genomes, prokaryotic virus genomes from NCBI RefSeq Virus database (v94) r2016 [86], and more than 12,000 viral genomes from the viral database PIGEON v1.0 [27]. ERW viral genomes were assigned into viral clusters (VCs) when clustering was significant (*p* < 0.05) and classified as outliers when clustering was non-significant. All unclustered viral genomes were classified as singletons.

For RNA viruses, previously identified ERW RdRP sequences and RdRP sequences from a custom database containing more than 613,000 RNA virus genomes from environmental metatranscriptomic studies and Refseq prokaryotic virus genomes (see above) were clustered using MMseqs2 v14 [73] with a sequence similarity threshold of 0.5 to identify clusters unique to ERW or shared with other datasets. To complement this gene-based clustering analysis, generalized unweighted UNIFRAC distances were calculated using *GUniFrac* package on R, with *α* = 0.5 (parameter controlling weight on abundant lineages) to evaluate the distance between datasets based on the sequences included in the RdRP phylogeny analyses described above.

### Viral genome annotation, activity, and infection cycle prediction

To evaluate DNA viral activity, metatranscriptomic libraries were used to identify expressed genes in each viral DNA genome. A gene was considered as expressed if the coding strand had ≥ 50% of its positions covered and > 0 median coverage depth [87] (Supplementary Fig. 2). DNA vOTUs for which at least one expressed gene was detected were classified as active. Based on the genome annotation described above, active DNA vOTUs that expressed genes related to virion structure, encapsidation, and/or lysis functions were classified as undergoing a lytic infection (“active—lytic”) while the other active DNA genomes were considered as possibly lysogenic or chronic infections and broadly classified as “active—other.” Additionally, for DNA vOTUs identified in known bacteriophage/archaeovirus taxa, BacPhlip v0.9.6 [88] was used to predict lifestyle (i.e., temperate or lytic) based on the vOTU representative sequences.

While the same approach of identifying expressed genes in metatranscriptomes is not possible for RNA viruses given their RNA-based genome, we still evaluated the activity of the dominant ssRNA *Lenarviricota* viruses using the strand-specific mapping information of metatranscriptomes. The rationale was that, while these genomes are expected to be single-stranded, their replication process involves the formation of a complementary negative strand [89]. Hence, RNA vOTUs with ≥ 50% of the genome covered at 1 × or more on the coding strand with a > 0 median coverage depth were considered as detected, while RNA vOTUs for which both strands were covered for ≥ 50% at 1 × or more were classified as detected and active (Supplementary Fig. 2).

### Statistical analyses

All statistical analysis and figures were performed in R (CRAN) [84] and Rstudio using the *vegan*, *ggplot2*, and *ComplexUpset* packages, and STAMP (Statistical Analysis of Metagenomic Profiles) software v 2.1.3 [90]. Non-metric multidimensional scaling (nMDS) ordination and hierarchical clustering analysis based on Bray–Curtis dissimilarity matrices, using the *vegan* package, was used to visualize sample comparisons. Bray–Curtis dissimilarity matrices were also generated for both DNA and RNA viral communities to visualize the similarity between and within months. Analyses of variance (ANOVA) and permutational multiple analysis of variance (PERMANOVA) tests were used to identify significant differences in viral community composition between dates, depths, and locations. Finally, we tested the significance of changes in active DNA vOTU abundance between months with a multiple group statistic test (ANOVA), a post hoc test (Tukey–Kramer) to identify which pairs of months differ from each other, and a multiple test correction (Storey’s FDR) to control false discovery rate, using STAMP. Post hoc plots generated by STAMP show the results of each significant test (corrected *p* value < 0.01) and provide an effect size measure for each pair of months. Based on the STAMP analyses, we then plotted the temporal dynamics (*z* score of RPKM) of each active DNA vOTUs exhibiting significant changes in abundance between months based on the “ecological strategy” of the assigned host, using the iPHoP analyses (see above). Based on Sorensen et al. [6], each host was associated to an “ecological strategy” depending on the month (or season) a given host was most abundant. All active DNA vOTUs without an assigned host or assigned to a host without a clear ecological strategy were plotted in a separated panel.

### Data availability

All available metagenomic and metatranscriptomic data are available through the IMG/M portal (assemblies) and NCBI SRA (reads). IMG/M and SRA identifiers of all metagenomes and metatranscriptomes, along with detailed information for each sample, are available in Supplementary Tables 1 and 2.

## Results

### High diversity of DNA and RNA phages in the East River Watershed soil dataset

To characterize the soil viral diversity in the ERW, we analyzed 46 and 43 paired metagenomes and metatranscriptomes, respectively, obtained from samples collected in hillslope locations at three depths over four dates from March 2017 to September 2017 (Fig. 1A, and Supplementary Table 1 and 2). Using established protocols combining virus sequence detection with VirSorter2 [63] refinement with CheckV [64], and clustering into non-redundant vOTU [85], we recovered 4047 and 5032 non-redundant DNA and RNA viral genomes, respectively (Supplementary Tables 3 and 4). As for previous studies of soil viruses via metagenomics, representative contigs from DNA vOTUs included a mix of (near-)complete genomes and genome fragments, with nearly 23% (907) longer than 10 kbp, and 101 identified as high-quality (> 90% complete) (Fig. 1B and C). Meanwhile, because RNA virus genomes are typically shorter, a larger proportion (*n* = 1870, 37%) were identified as high-quality (> 90% complete) genomes (Fig. 1B and D).

**Fig. 2:**
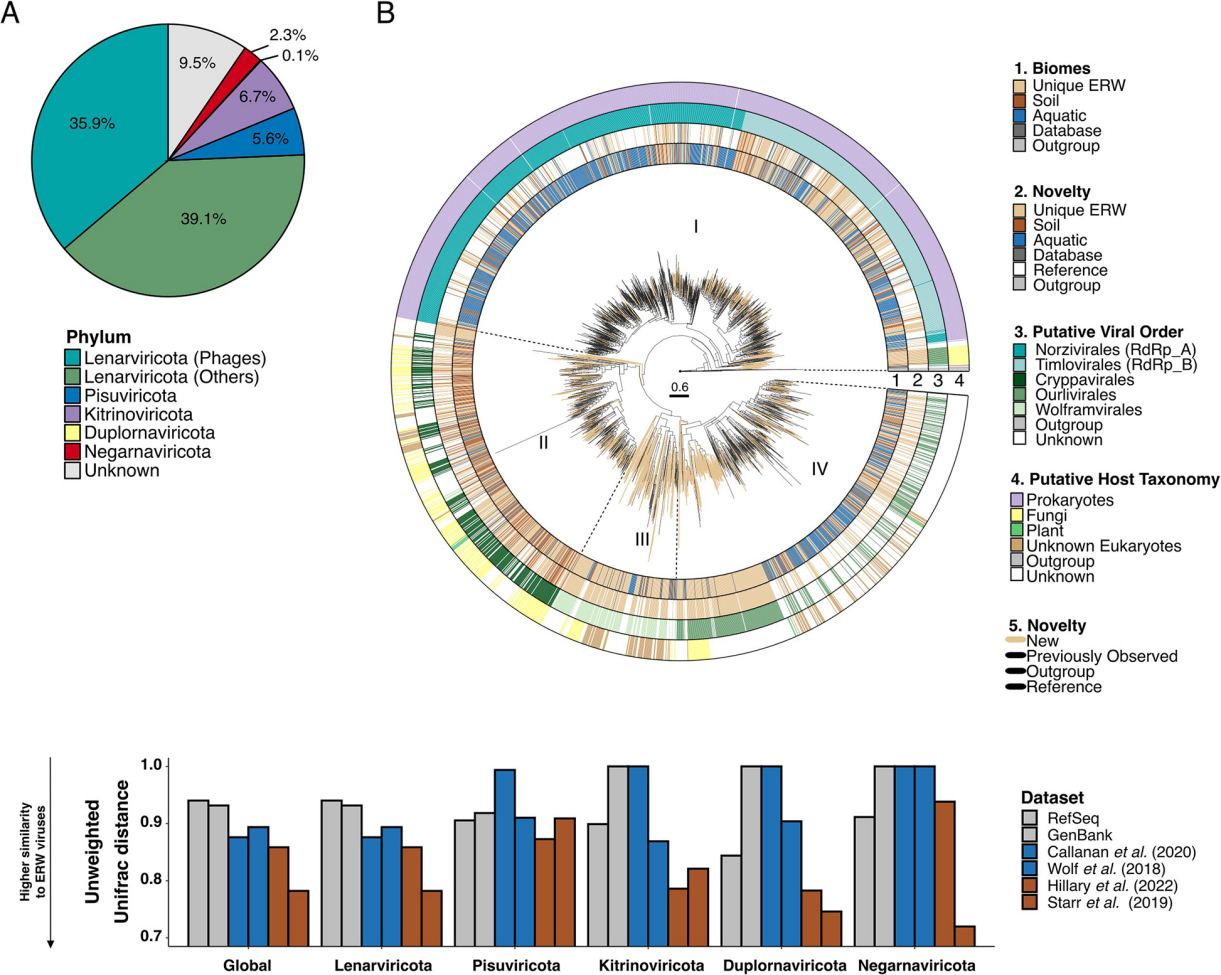
Diversity and phylogenetic analyses of ERW RNA viral communities.** A** Distribution of RNA viral phyla across ERW RNA virus sequences, based on taxonomic assignments from RdRP phylogenies. The Lenarviricota phylum is further divided between bacteria-infecting viruses (“phages,” *Leviviricetes*) and eukaryote-infecting viruses. **B** Rooted phylogenetic tree of RdRP sequences belonging to the ssRNA Lenarviricota phylum. The tree is rooted using reverse transcriptases as an outgroup and visualized with ggtree. The tree contains 1331 cluster representatives from ERW soil samples (ring 1, light brown), aligned with those used to construct the RNA global phylogeny from previous metatranscriptomic studies and public databases. Clusters composed exclusively of ERW sequences are colored in brown (ring 2) with branches leading to these clusters highlighted in light brown in the tree, while clusters composed of ERW sequences and existing virus sequences are colored by the environment type of the study (soil: dark brown, aquatic: blue, public databases: dark gray). Virus taxonomy (ring 3) and host (ring 4) are predicted based on the position of reference sequences from the RefSeq database in the tree (see the “Methods” section). **C** Unweighted UNIFRAC distances between RdRP sequences identified in this study and previously published collections of environmental RNA viruses [14, 29, 78, 80]. ERW and Starr sequences were obtained from RNA shotgun sequencing of bulk samples, while Hillary sequences come from RNA shotgun, sequencing of viral fraction (see [14]). UNIFRAC distances were calculated and are presented separately for each of the 5 RNA virus phyla, with the average distance presented in the first “Global” column. Reference databases are colored in gray, studies from aquatic environments in blue, and soils in dark brown. A distance close to 0 means that the two datasets are phylogenetically similar

A marker gene taxonomic classification performed using GeNomad [76] suggested that the vast majority (> 90%) of classified DNA vOTUs were bacteriophages from the *Caudoviricetes* class, i.e., tailed dsDNA bacteriophages typically identified in soil metagenomes [26] (Fig. 1C and Supplementary Table 3). Meanwhile, ~ 50% of RNA vOTUs were classified into the *Leviviricetes* bacteriophage class, while the remaining 44.6% of vOTUs were assigned across all five recognized phyla of RNA viruses (i.e., *Lenarviricota* outside of the *Leviviricetes* class, *Pisuviricota*,* Kitrinoviricota*,* Duplornaviricota*, and *Negarnaviricota*) (Fig. 1D and Supplementary Table 4). About 5% of RNA vOTUs were not assigned to known RNA virus phyla. To refine this marker-based affiliation of RNA viruses, we performed a phylogenetic analysis of RNA viruses based on the RdRP marker gene (RNA-dependent RNA polymerase) [14, 28, 29, 78]. After clustering ERW RdRP sequences with references and previously published datasets obtained from soil metatranscriptomes at 50% average amino acid identity (AAI) [28, 91], each cluster representative was assigned to a phylum, and a phylogeny was built for each phylum. Consistent with the marker gene geNomad classification, most of the ERW RdRP grouped within the positive-sense single-stranded *Lenarviricota* (75%), followed by the *Kitrinoviricota* (6.7%) and *Pisuviricota* (5.6%) (Fig. 2A). The phylogenetic tree of phylum *Lenarviricota* could be further divided into four subclades; consistent with previous evolutionary studies of this phylum [92], the first one corresponds to sequences branching within the *Leviviricetes* class (35.8%), the second and third to sequences branching next to the *Ourlivirales* (11.6%) and *Cryppavirales* (8.8%) orders, while the fourth group represented novel clade with no closely related sequence within the *Wolframvirales* (4.5%) order (Fig. 2B and Supplementary Table 5). Among the other phyla, *Picornavirales* (2.5%),* Tolivirales* (2.3%), and *Martellivirales* (1.3%) were the most represented orders (Supplementary Fig. 3A to D and Supplementary Table 6 to 9). Taken together, these results highlight the high diversity of both DNA and RNA viral communities in ERW, with an unsuspected high richness of RNA phages in ERW soils.

### Ecological distribution of ERW soil virus diversity and host connections

We next compared this ERW viral diversity to reference viruses and soil virus metagenomic datasets using either genome-based (vContact2 [85], for dsDNA phages >  = 10kbp) or marker gene-based clustering (RdRP, 50% AAI, for RNA viruses). While these analyses included > 12,000 soil DNA phages and 613,000 RNA virus sequences, 20% of the ERW dsDNA phages (Supplementary Table 10) and 37% of the ERW RNA viruses were found in clusters composed only of ERW samples (Fig. 1C and D). Another 36.4% (for DNA phages) and 41% (for RNA viruses) were only clustered with other metagenome-derived virus genomes sampled from soil- and/or plant-related samples, and only 11 DNA and 220 RNA vOTUs were clustered with viruses from the RefSeq database (Fig. 1C and D). In particular, the majority of ERW vOTUs assigned to the RNA virus orders *Wolframvirales* (98.0%), *Ourlivirales* (84.2%), and *Cryppavirales* (56.4%) within the *Lenarviricota* phylum were found in ERW-specific clusters (Supplementary Table 4), suggesting that the diversity of soil viruses within these three families may be largely under-characterized (Fig. 2B). Overall, these results indicate that the ERW phages and viruses are mostly novel compared to isolated references, but display some similarity to other uncultivated soil viruses, consistent with the existence of a “global soil virosphere” which is still only partially sampled [27]. This was confirmed for RNA viruses via UNIFRAC analyses of the different phylum-wide phylogenies, which indicated that ERW sequences overall were more closely related to sequences from other soil samples rather than sequences from other environments or references (Fig. 2B and C, and Supplementary Table 11).

Applying a new integrated phage-host prediction method (iPHoP [77]) which relies on an ensemble of phage-based and host-based approaches, 1529 (37.8%) DNA vOTUs could be associated with a host genus or family (Supplementary Fig. 4 and Supplementary Table 3). Most of the predicted hosts were assigned to *Actinomycetia* (*n* = 618, 15.3%), *Alphaproteobacteria* (*n* = 506, 12.5%), and *Gammaproteobacteria* (*n* = 92, 2.27%), representing ~ 80% of the total predicted hosts (Supplementary Fig. 4). On the other hand, the same approach (iPHoP) did not yield reliable host predictions for most RNA viruses, as this tool was primarily designed for DNA bacteriophages and archaeoviruses. Instead, we leveraged the RdRP phylogenies (see above) to identify putative hosts for RNA viruses, especially between bacteria and major divisions of eukaryotes. Overall, 2449 (48.7%) RdRP branched within the *Leviviricetes* class and were assigned to prokaryote hosts, along with the 9 RdRP branching within the *Cystoviridae* family (Fig. 1D, and Supplementary Table 4). 723 RdRP branched within clades associated with fungal hosts (14.4%) (Fig. 1D and Supplementary Table 4**)**, including 31 (59.6%) (Supplementary Fig. 3C) and 41 (36.0%) (Supplementary Fig. 3D**)** clades in the *Duplornaviricota* and *Negarnaviricota* phyla, respectively. Finally, the rest of the RdRP were found in clades associated with various eukaryotic hosts (30%), or without any isolate representative (6.8%), highlighting the vast extent of soil RNA virus diversity still to be characterized.

### Contrasted dynamics of DNA and RNA viruses across seasons

**Fig. 3:**
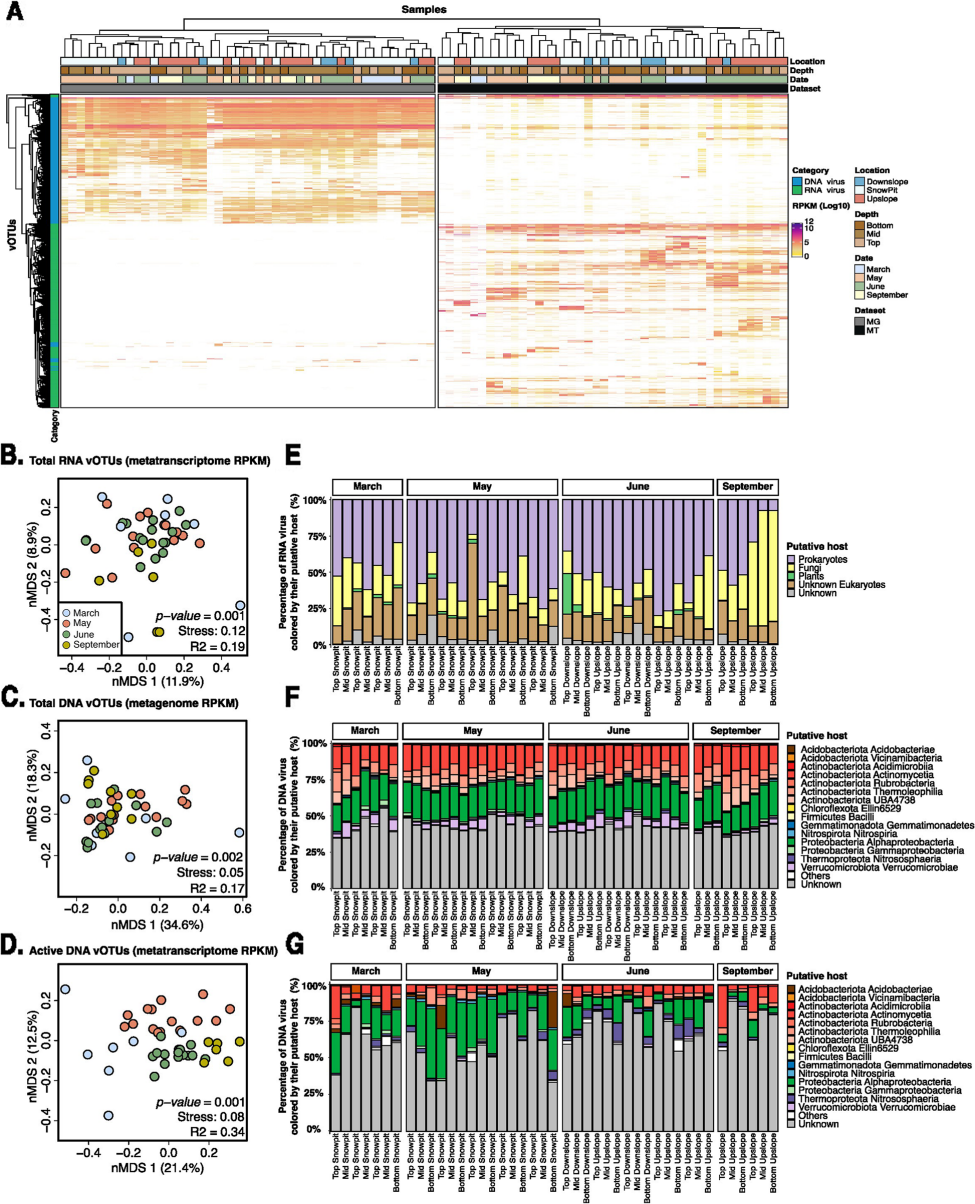
Overview of the temporal dynamics of total and active DNA and RNA viral communities.** A** Distribution of all DNA (blue) and RNA (green) vOTUs across metagenome and metatranscriptome datasets. The vOTUs and samples are clustered based on vOTU relative abundances (log-transformed RPKM). Color bars above the heatmap indicate the location, depth, season, and type of each dataset. The left bar (category) indicates the vOTUs which are classified as DNA or RNA virus. **B–G** Beta-diversity of total RNA (***B***), total DNA (***C***), and active DNA (***D***) viral communities across the 4 seasons. For each group of viruses, non-metric multidimensional scaling (NMDS) ordination plots, representing the (dis)similarities between samples based on vOTU relative abundance, are presented in the left panels (***B***, ***C***, and ***D***). Individual samples are colored based on season: September (yellow), March (blue), May (red), and June (green). Stress values associated with two-dimensional ordination and PERMANOVA results describing the variance in community composition explained by season are reported for each plot. The RPKM of total RNA (***E***), total DNA (***F***), and active DNA (***G***) vOTUs predicted to infect putative host groups (for RNA vOTUs) or bacterial class (for DNA vOTUs) is indicated for each. “Other” represents the remaining host classes (representing less than 0.1% of hosts). For “active” DNA, only vOTUs identified as active were considered (see the “Methods” section), and the RPKM from metatranscriptome read mapping was used as an estimation of the relative abundance instead of RPKM from metagenome read mapping

We next investigated the dynamics of both DNA and RNA viral communities across seasons, depths, and locations, using both presence/absence and nMDS ordination analyses based on estimated relative abundances (RPKM). Overall, 2758 (68.2%) and 1238 (24.6%) DNA and RNA viruses, respectively, were found at least in one sample of each season (Fig. 3A). Conversely, 373 (9.2%) and 1208 (24%) DNA and RNA viruses, respectively, were only found in a specific season (Supplementary Fig. 5A and B), indicating that both communities may exhibit seasonal dynamics, although of different magnitude. These patterns were confirmed by nMDS ordination analyses showing that both RNA (PERMANOVA; *R*^2^ = 0.19; *p* < *0.001*) and DNA (PERMANOVA; *R*^2^ = 0.17; *p* < *0.01*) viral community differed significantly by season (Figs. 3B and C), while depth had only a marginal effect on the DNA viral community (PERMANOVA; *R*^2^ = 0.11; *p* < *0.01*) and soil location had no significant effect on both communities (PERMANOVA; *p* > *0.05*) (Supplementary Table 12). Bray–Curtis dissimilarities between months across successive seasons were also systematically significantly higher for the RNA viral community compared to the DNA viral community, suggesting that RNA viruses underwent a higher rate of turnover between seasons (ANOVA; *p* < *0.001*) (Supplementary Fig. 5C). Finally, given the relative “stability” of the DNA virus community observed in metagenomes, we performed the same analysis for DNA vOTUs based on RPKM from metatranscriptomes, reasoning that transcriptional activity rather than relative abundance from metagenomes may uncover stronger seasonal patterns. Indeed, an nMDS ordination based on DNA vOTU metatranscriptome RPKM was strongly structured by season (PERMANOVA; *R*^2^ = 0.34; *p* < *0.001*) (Fig. 3D). Altogether, these results indicate that both DNA and RNA virus communities are dynamic throughout the year, reflected primarily by a strong turnover for RNA viruses and changes in terms of which subset of the community is transcriptionally active for the dsDNA phages.

Some of these seasonal dynamics can be further explained by grouping vOTUs based on their predicted hosts. Throughout the year, the RNA virus community was characterized by an increased relative abundance of phages (mainly *Leviviricetes*;* Timlovirales*) in May during the snowmelt period, followed by an increase in relative abundance for plant-infecting viruses in June when the perennial plants emerge from dormancy and for fungi-infecting viruses (*Cryppavirales*) in September (Fig. 3E and Supplementary Table 13) following plant senescence and litter accumulation. For DNA phages, only a small number of phages predicted to infect *Thermoleophilia*, *Chloroflexota Ellin6529*, *Vicinamibacteria*, and *Verrucomicrobiae* bacterial hosts displayed a significant seasonal turnover (ANOVA; *p* < *0.001*) (Fig. 3F and Supplementary Table 13). Meanwhile, other groups of DNA phages found in both metagenome and metatranscriptome samples showed a significant seasonal dynamic based on their metatranscriptome RPKM, with a higher proportion of phages predicted to infect *Alphaproteobacteria*,* Acidimicrobiia*,* Verrucomicrobiae*,* Nitrospiria*, and *Actinomycetia* showing the highest change in activity over season (ANOVA; *p* < *0.001*) (Fig. 3G and Supplementary Table 13). These suggested that different types of virus-host interactions and ecological successions coexist in ERW soils with different ecological impacts.

### Increased activity of DNA and RNA viruses after snowmelt

**Fig. 4:**
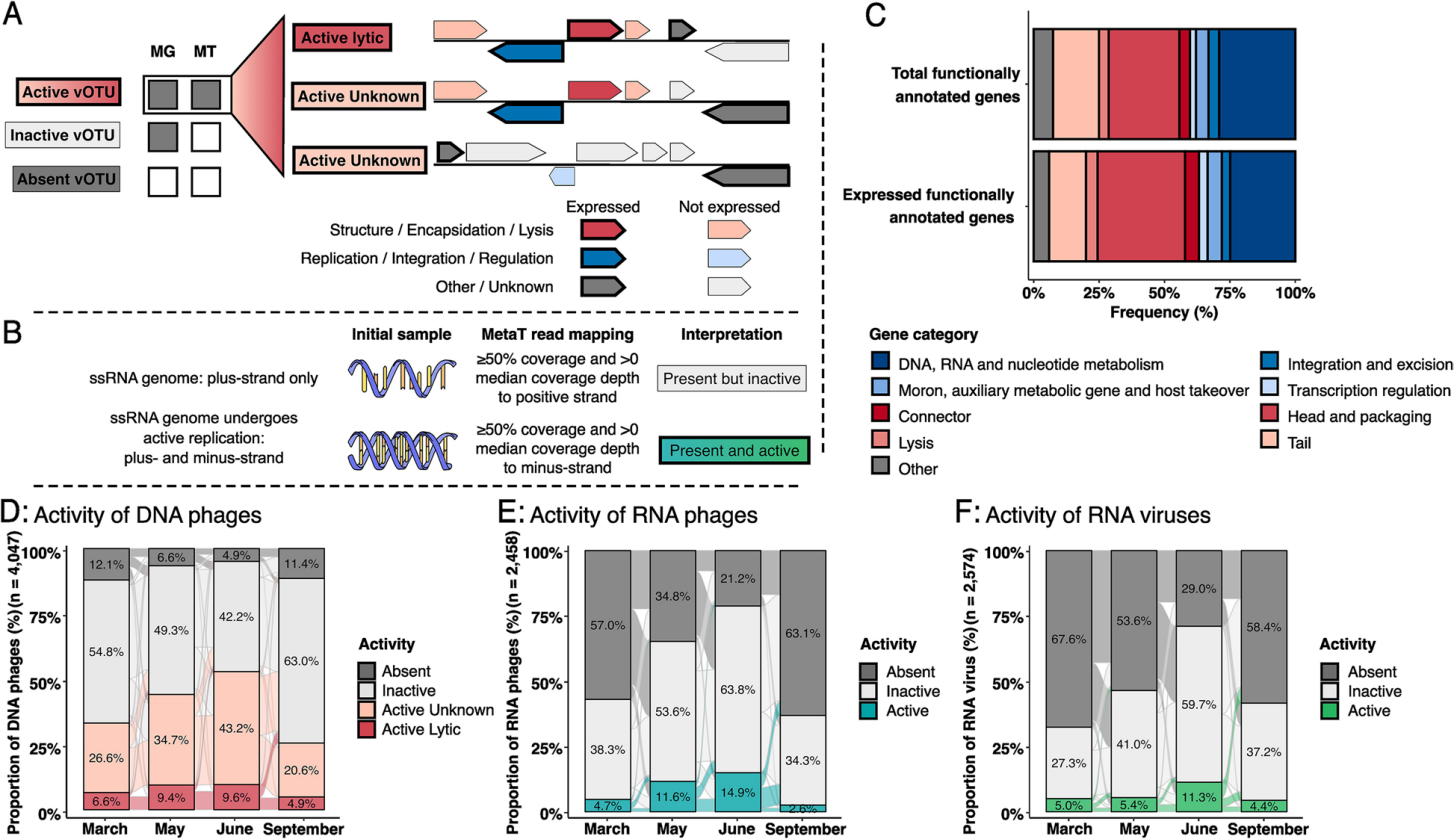
Functional annotation and activity of DNA and RNA viruses. **A** Schematic representation of the framework used for assessing the activity, including the infection stage, of DNA phages. Metatranscriptome read mapping is used to identify expressed genes in each viral DNA vOTU, and the number and annotation of these genes are then used to determine the activity and the infection stage of each DNA vOTU. DNA phages for which at least one expressed gene was detected were classified as active (Supplementary Fig. 2). **B** Schematic representation of the framework used for assessing the activity of ssRNA viruses. Based on metatranscriptome read mapping, ssRNA viruses are classified as actively replicating if both coding and non-coding genome strands are detected and considered as “present” if only the coding strand is detected. **C** Proportion of total and expressed annotated genes based on functional annotation using Prokka v1.14.6 from DNA viral genomes by aligning them against PHROGS v4 database, with an *e* value cutoff 1E − 6. Functional categories associated with lytic infections, i.e., categories associated with virion production and host cell lysis, are colored in red, and the other major phage functional categories are colored in blue. Only genes that were annotated are included in the figure, and the proportion of annotated genes over all genes in the (active) DNA vOTUs is indicated next to each bar chart. **D** Proportion of active (dark and light red), inactive (light gray), and absent (dark gray) DNA phages across months. Within DNA vOTUs identified as active, the ones likely engaged in active lytic infection were identified based on the functional annotation of expressed genes, while other active vOTUs are identified as “active—unknown.” The spaces between the different branches represent the transition (or connection) of each vOTU between categories. **E**,** F** Proportion of active (blue and green), inactive (light gray), and absent (dark gray) RNA phages (**E**) and RNA viruses (**F**) across months. A vOTU is considered as active when it is detected as active in at least one sample. Transcriptional activity is measured by read mapping from at least one metatranscriptome to at least one gene of the vOTU (Supplementary Fig. 1). The proportion of active vOTUs for each month is the sum of all active vOTUs for a given month

Because the viral populations were detected from bulk soil samples, they presumably represent a mixture of proviruses (i.e., integrated or extrachromosomal viruses that reside within their host cell), actively replicating viruses, and some extracellular viral particles [9]. Metatranscriptomes offer a unique opportunity to further understand which soil viruses are active, where, and when. To evaluate DNA viral activity at the vOTU level, metatranscriptomic libraries were used to identify expressed genes in each viral DNA genome, and viruses for which at least one expressed gene was detected were classified as active (Fig. 4A). Among these, viral genomes that expressed genes related to virion structure, encapsidation, and/or lysis functions were classified as undergoing a lytic infection. The same approach is not possible for RNA viruses given their RNA-based genome; however, for the dominant RNA viruses (the ssRNA *Lenarviricota* phylum), we instead leveraged strand-specific mapping information to identify actively replicating viruses based on the detection of both coding and non-coding genome strands (Fig. 4B). This enabled a comparative analysis of activity levels for DNA and RNA viruses in ERW.

Overall, 8937 genes (31.5%) were identified as expressed across 3106 DNA viral genomes, including 1101 genes with functional annotation (Fig. 4C and Supplementary Table 14). A total of 2926 (72.3%) DNA viral vOTUs were detected as active in at least one sample, and 535 (13.2%) were classified as active lytic viruses based on functional annotation (Fig. 4D**)**. Meanwhile, for RNA viruses among the *Lenarviricota* phylum, 24.5% (600 vOTUs) of the *Leviviricetes* (Fig. 4E) and 18.7% (294 vOTUs) of the eukaryote-infecting viruses (Fig. 4F) were detected as active in at least one sample. Across seasons, the overall proportion of active DNA viruses significantly increased from March (33.2%) to June (52.8%), and decreased in September (25.5%) (PERMANOVA; *R*^2^ = 0.352; *p* < *0.001*) (Supplementary Table 12). Both temperate and virulent DNA phages showed significant increases in transcriptional activity from March to June** (**Supplementary Fig. 6A and B). For temperate phages however, the fraction of vOTUs associated with an active lytic infection, i.e., expressing one or more lysis-related genes, was stable from March to June (Supplementary Fig. 6A). For temperate phages, it is thus possible that the increased detection of transcriptional activity is at least partially due to gene expression from prophages as part of lysogenic cycles and not to induced prophages. A similar overall pattern was recovered for both RNA bacteriophages (*Leviviricetes*) and other eukaryote-infecting *Lenarviricota*, with significant increases in activity from March (4.7% and 5.0%) to June (14.9% and 11.3%) (PERMANOVA; *R*^2^ = 0.214; *p* < *0.001*) (Supplementary Table 12), suggesting that similar large-scale seasonal variations, here in particular the snowmelt followed by subsequent plant growth season, likely shape the activity of both DNA and RNA viral communities. Within this overall increase however, the highest increase in numbers of both active DNA and RNA phages occurred between March and May (peak snow-to-snowmelt period), while the largest increase in active RNA eukaryote-infecting viruses occurred between May and June (plant emergence post snowmelt), suggesting a delayed response to snowmelt for some eukaryotic viruses compared to phages.

### Active phages are connected with the bacterial community structure in ERW soils

The increased activity observed for DNA phages through summer (i.e., from March to June), combined with the strong seasonal effect observed from metatranscriptome RPKM but not metagenome RPKM for the same DNA phages suggest that the ERW DNA phage community may be structured as a “seed-bank.” This seed-bank is likely composed of a large group of persistent and mostly inactive phages residing in soils or in their host, and a different subset actively replicating and lysing their host across seasons, in particular following and/or concomitant with host growth/bloom.

**Fig. 5:**
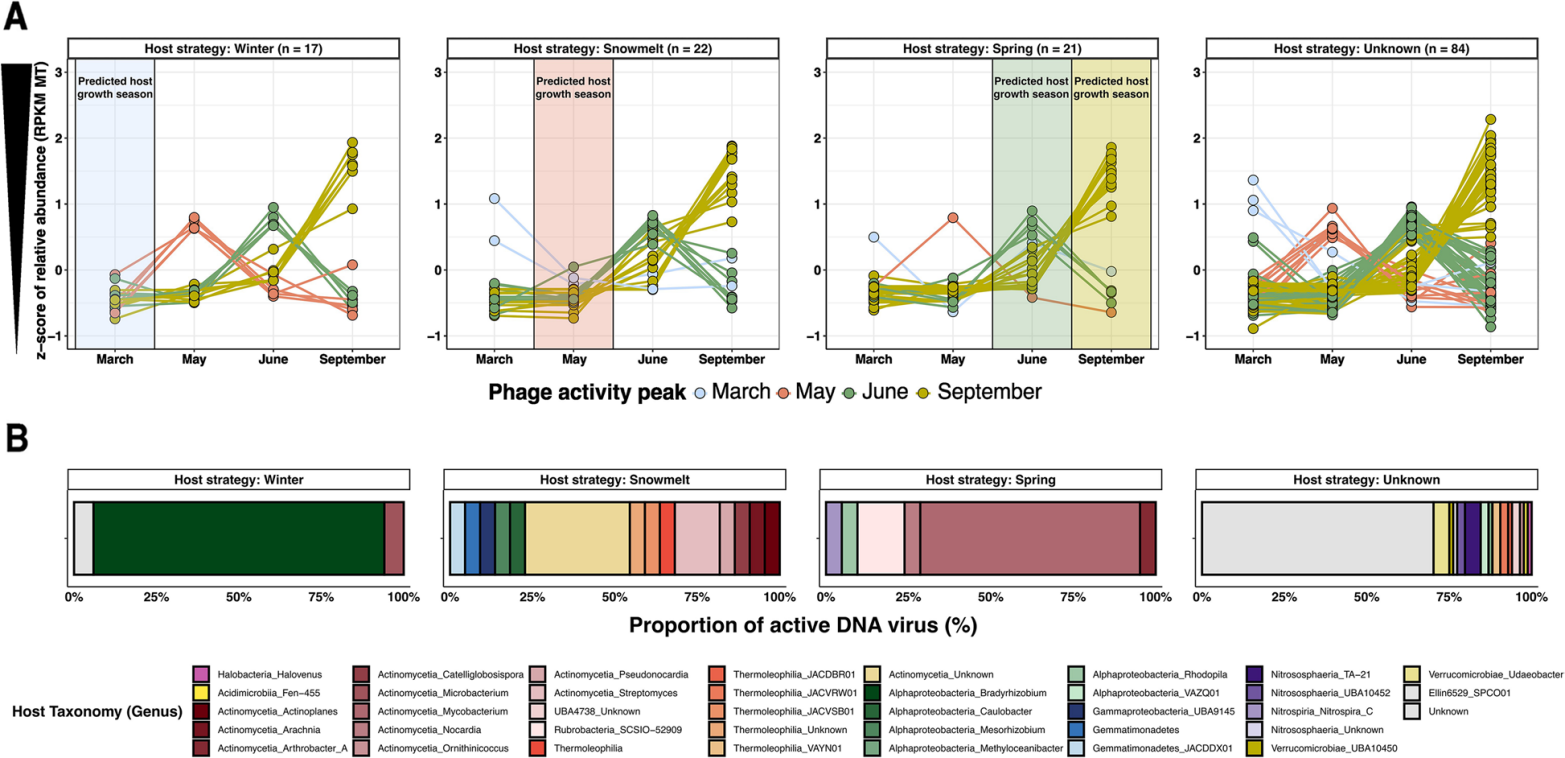
Connected temporal dynamics of predicted virus-host pairs.** A** Temporal dynamics of active DNA phages exhibiting a significant seasonal pattern in activity (*n* = 144). The significance of changes in abundance between months was tested with a multiple group statistic test (ANOVA), a post hoc test (Tukey–Kramer) to identify which pairs of months differ from each other and a multiple test correction (Storey’s FDR) to control false discovery rate, using STAMP. The seasonal dynamic of each active DNA vOTU exhibiting significant changes in abundance between months was plotted using the mean of metatranscriptomic RPKM transformed in *z*-score. Each vOTU was associated to a specific season based on its peak of activity (colored lines). vOTU dynamics are grouped by panel depending on the “ecology strategy” of their assigned host (see the “Methods” sections). Each host was associated to an “ecological strategy” depending on the month (or season) a given host was supposed to be growing [6], represented by colored boxes in each panel. Finally, all active DNA vOTUs without an assigned host or host without a clear ecological strategy were plotted in the last panel. **B** Distribution of predicted host taxa for active DNA vOTUs with significant seasonal activity pattern (see above), grouped by host growth strategy as in panel **A**

To better characterize the relationship between host growth and viral activity, we first used similarity percentage breakdown (SIMPER) [93] analyses to identify which DNA phage vOTUs were differentially active across seasons. Overall, 144 (5%) active DNA phages exhibited significant and clear differential abundance patterns in metatranscriptomes across seasons (ANOVA, effect size > 0.3, adjusted *P* < 0.05) and could each be associated to a specific “high activity season” (Fig. 5A and Supplementary Table 15). The remaining 95% of DNA phages typically exhibited significant differential abundance across samples, but were too variable between samples within each month to be associated with a clear and systematic seasonal pattern (Supplementary Fig. 7A and B). We next investigated whether, for the subset of viruses with clear seasonal activity pattern, their peak of activity may be connected to their host growth dynamics. Overall, 41.7% of these representative phages were assigned to a host taxon that was previously associated by Sorensen et al. [6] to a specific ecological strategy (i.e., Winter-adapted, Snowmelt-specialist, and Spring-adapted taxa), which allowed us to explore the dynamics of active phage-host interactions across seasons (Fig. 5B and Supplementary Table 16). In a somewhat counter-intuitive manner, DNA phages infecting both winter-adapted and snowmelt-specialist bacteria were least active when their predicted bacteria host would be growing, i.e., in March and May, respectively (Fig. 5A). Conversely, DNA phages infecting spring-adapted bacteria were most active during the expected growth period of their hosts, between June and September. Phage-host interactions in the ERW thus appear to follow at least two distinct patterns: for the limited diversity of bacteria adapted to “slow” growth under snow or immediately upon snowmelt, the majority of DNA phage activity seems to be delayed compared to this initial seasonal growth of the host. On the other hand, the growth of a diverse community of bacteria in Spring following snowmelt seems to be associated with concomitant DNA phage activity. This suggests that, for viruses with strong seasonal activity patterns, the optimal infection strategy may be, at least partially, driven by the ecological and growth strategy of their host. Complementarily, DNA phage activity may also respond directly to soil temperature variations across seasons, as soil temperatures may be near or above 0 °C under snow cover and jump to 4 °C in a short time period during the snowmelt period [6].

## Discussion

Leveraging a dataset of paired metagenomic and metatranscriptomic libraries from mountainous soil samples across seasons, we identified thousands of DNA and RNA viruses and assessed their diversity, community structure, and activity patterns. These data contribute to a better characterization of soil virus phylogenetic and genomic diversity and suggest that soil virome composition and activity seem to be affected by both their host metabolism and by ecological features of their local sampling environment, especially variation across seasons and, to a lesser extent, soil depth. The ERW soils sampled in this study were characterized by a dominance of bacteriophages with two types of population: a large group of inactive phages residing in soils or in their hosts and a smaller group of (often temporarily) active phages. The persistence of inactive viruses across seasons could have been facilitated by the continuous presence of putative hosts [30] and/or low soil temperatures preventing viral inactivation [94], especially under snow. Alternatively, it is possible that some of these persistent viruses maintain a low level of activity throughout the year not detectable in the current data. Regardless of the underlying mechanism of persistence, these phages likely represent a “seed-bank” from past lytic events that may serve as a reservoir for new infections to emerge when conditions become advantageous [95]. Given the abundance and diversity of hosts infected by these active viruses, the proliferation of these active and lytic phages will very likely have substantial impacts on the microbial communities and thus on the soil biogeochemical cycling.

Phages are becoming increasingly recognized for their essential ecological roles, especially as they can control host population dynamics [31, 46, 96]. During lytic phases, virulent phages invade, replicate in, and eventually kill their hosts, which can result in a substantial reduction in the relative abundances of the dominant microbial community members, as illustrated by the “Kill-The-Winner” (KTW) model [12, 13, 97–100]. In contrast, temperate phages, which are considered to be fairly common in soil [21, 33, 101, 102], have an opportunity to reside in their hosts via lysogenic infections rather than lysing them. This led to alternative virus-host dynamic models, such as for instance “Piggyback-The-Winner” (PTW), which predicts that phages integrate into their hosts’ genomes as prophages when microbial abundances and growth rates are high [96]. Finally, the factors determining the lytic to lysogenic switch in complex soil microbial communities remain poorly understood. In the ERW soils, lytic infection of active hosts seemed favored in spring and early summer (May/June), consistent with KTW interactions. This model is expected to occur under favorable conditions, such as a nutrient enrichment or wetting soils, generating high bacterial diversity [30] and favoring lytic over lysogenic infections [103]. Such conditions are consistent with environmental conditions during the plant-growing season in ERW soils, when ammonia-oxidizing Thaumarcheota are highly abundant and active [6], perhaps due in part to nutrient enrichment caused by phage-induced microbial host lysis. Conversely, the temporal delay between the activity of phages and the growing period of their hosts under snow conditions or upon snowmelt associated with the low diversity of hosts may be best described by a third model, recently proposed: “Piggyback-The-Loser” (PTL) [44] or “Piggyback-The-Persistent” (PTP) [43], that suggests the opposite of the PTW model. This hypothesis argues that when the host is growing slowly, it is more beneficial to remain in a lysogenic state, as it is less likely that the phages produced through lysis will find a new host [45]. For example, we have previously shown that the microbial population size is smaller at peak winter snowpack depth in March compared to the substantial microbial biomass production that occurs during snowmelt in May, perhaps due to substrate preferences and growth rates that lead to niche partitioning among microbial hosts. Similarly, PTL or PTP was described in some regions of the deep sea or polar marine regions [44, 104], and the lower activity of DNA phages when their hosts are growing in ERW during March and May suggests that comparable infection strategies also occur in soils. Furthermore, the sequential identification of different infection dynamics across seasons suggests that environmental conditions and host growth strategies may likely be an important factor driving the selection for one viral strategy or the other.

In terms of community diversity, while only a few RNA bacteriophages have been experimentally characterized so far, we found a large number of RNA phages belonging to the *Leviviricetes* class in the ERW soils, including potentially novel phages associated with a wide range of bacteria inhabiting soils. In combination with recent investigations of *Leviviricetes* in terrestrial ecosystems [14, 28, 87, 91], our study emphasizes the understudied nature of RNA phages in soils in comparison to DNA phages. This can be explained by the difficulties to recover RNA from soil because it is more easily degraded than DNA and/or because RNA viruses are not detected in metagenomes. We also identified a significant part of the RNA phage community as actively replicating, and given that all known RNA phages are virulent, we hypothesize that these significantly contribute to overall bacterial death in ERW [46]. By partially driving the turnover of bacteria, the diverse, abundant, and active RNA phages recovered in this study are expected to impact soil microbiomes and associated biogeochemical cycling. Nevertheless, because of the limited availability of host predictions for RNA phages and the difficulties in linking them to even a high-order host taxon (e.g., a bacterial phylum or class), their impact on host communities in soil ecosystems remains difficult to evaluate more precisely. Further studies of global RNA phage diversity along with the development of new model systems, especially the cultivation of both RNA phages and their hosts, are now critical to understand the impact of these RNA phages on soil ecology [28, 105].

Finally, beyond the apparent dominance of ssRNA *Leviviricetes* bacteriophages in ERW soils, our phylogenetic analysis suggests that a significant number of RNA viruses detected in this work infect fungal, plant, and animal hosts. Among these, most of them display some similarity to other uncultivated soil viruses, with no match with isolated references, consistent with the existence of a “global soil virosphere” still only partially sampled [21, 27]. Interestingly, most of these viruses were predicted to infect fungal hosts, suggesting that the diversity of these mycoviruses may also be largely under-characterized [106]. Like the DNA and RNA phages, the eukaryotic-infecting RNA viruses seem to broadly follow their host dynamics, exhibiting a delayed peak of activity in comparison to phages. Because spring-adapted soil fungi at the East River field location are predominately mycorrhizal fungi [6], the interactions between eukaryotic-infecting RNA viruses and their fungal hosts may also influence ecosystem nutrient uptake and retention by affecting the relationship between mycorrhizal fungi and the plant roots which they colonize. These results suggest that RNA viruses are an integral component of global soil ecosystems, with a diversity and activity driven by the growth of their hosts and the environmental parameters which affect them [107]. The detection of expressed genes related to lysis functions, combined with an increase in this activity from Spring to Summer, suggests that viral lysis may influence the global availability of nutrients and organic matter in the ERW soils, as recently shown in microcosm experiments [108], especially in periods of high microbial activity and growth. Meanwhile, for other vOTUs, the specific expression of genes not involved in genome replication, capsid formation, or lysis would be consistent with an active reprogramming of their host metabolism outside of (or prior to) active virion production. With many vOTUs only represented by partial genomes and many unannotated genes, it is difficult to draw a stronger conclusion on this front; however, it is becoming increasingly clear that, in order to improve our understanding of the ecological roles of viruses in soil, both host cell lysis and host cell reprogramming will need to be considered and further characterized especially in the context of global nutrient cycling and carbon sequestration. 

## Conclusions

Altogether, our work reveals the temporal dynamic of viral activity and its possible connections to virus-host interactions that occur at different time scales. These interactions are influenced by various factors such as host strategies, viral resistance, and virus-host coevolution [109]. While these results are based on a single year of sampling, and further studies will be needed to verify that these strong seasonal trends persist across multiple years, these already highlight the necessity of incorporating more comprehensive models of host-virus interactions when characterizing and modeling viral diversity, activity, and the impact on hosts within soil ecosystems.

### Supplementary Information

**Additional file 1: Supplementary Fig. 1.** Overview of the Pipeline for ERW Soil Sample Analysis. **Supplementary Fig. 2.** Distribution of coverage breadth and coverage depth in metatranscriptomes for DNA and RNA vOTUs. **Supplementary Fig. 3.** Diversity and phylogenetic analyses of ERW RNA viral communities. **Supplementary Fig. 4.** Completeness, global distribution, virus taxonomy, and host taxonomy for all DNA vOTUs (< 10 kb +  ≥ 10 kb). **Supplementary Fig. 5.** Temporal dynamics of total and active DNA and RNA viral communities. **Supplementary Fig. 6.** Activity of DNA phages. **Supplementary Fig. 7.** Temporal dynamics of non-significant virus-host relationships.

**Additional file 2: Supplementary Table 1**. Description of the 46 IMG metagenomes and 43 metatranscriptomes (related to Fig. 1A). **Supplementary Table 2.** Metadata for all the East River Watershed soil samples (related to Fig. 1A). **Supplementary Table 3**. DNA vOTU table with RPKM for each vOTU by sample, contig information, taxonomy and host prediction (related to Fig. 1C). **Supplementary Table 4**. RNA vOTU table with RPKM for each vOTU by sample, contig information, taxonomy and host prediction (related to Fig. 1D). **Supplementary Table 5**. Detailed information of RNA vOTUs linking RdRP Set 1 (related to Fig. 2). Viral taxonomy is based on Coat protein (CP) from Callanan et al., (2020)  [80] and RdRP marker genes. "Putative_Viral_order", "Putative_Viral_family", "Putative_host" columns are based on predictions made with a custom script (See Materials). **Supplementary Table 6**. Detailed information of RNA vOTUs linking RdRP Set 2 (related to Suplementary Fig. 1). "Putative_Viral_order", "Putative_Viral_family", "Putative_host" columns are based on predictions made with a custom script (See Materials). **Supplementary Table 7**. Detailed information of RNA vOTUs linking RdRP Set 3 (related to Suplementary Fig. 1). "Putative_Viral_order", "Putative_Viral_family", "Putative_host" columns are based on predictions made with a custom script (See Materials). **Supplementary Table 8**. Detailed information of RNA vOTUs linking RdRP Set 4 (related to Suplementary Fig. 1)."Putative_Viral_order", "Putative_Viral_family", "Putative_host" columns are based on predictions made with a custom script (See Materials). **Supplementary Table 9**. Detailed information of RNA vOTUs linking RdRP Set 5 (related to Suplementary Fig. 1). "Putative_Viral_order", "Putative_Viral_family", "Putative_host" columns are based on predictions made with a custom script (See Materials). **Supplementary Table 10**. Output table generated by vConTACT2 analysis with all the taxonomic information to reference genomes, as well as all the clustering information (related to Fig. 1C). **Supplementary Table 11**. UNIFRAC distances between this study and environmental studies (related to Fig. 2C). “Hillary” dataset was described in Hillary et al., (2022) [14], “Starr” was described in Starr et al., (2019) [29], "Callanan" was described in Callanan et al., (2020) [80], and "Wolf" was described in Wolf et al., (2020) [79]. **Supplementary Table S12**. PERMANOVA testing of contribution of season, depth and location on total DNA and RNA viral community and active DNA and RNA viral community (related to Fig. 3BCD) **Supplementary Table 13**. Significance of changes in total and active DNA and RNA vOTU abundance between months by ANOVA and post-hoc tests (related to Fig. 3EFG). **Supplementary Table 14**. Functional annotation of protein families against PHROGS database (related to Fig. 4C). **Supplementary Table 15**. Information (RPKM for each vOTU by sample, contig information, taxonomy and host prediction) of active DNA vOTUs exhibiting significant changes in abundance (*n* = 144) determined by STAMP analyses presented in Table S13 (related to Fig. 5A). **Supplementary Table 16**. Ecological strategies assigned to archaeal and bacterial OTUs from Sorensen et al., (2020) [6].

## Data Availability

All available metagenomic and metatranscriptomic data are available through the IMG/M portal (assemblies) and NCBI SRA (reads). IMG/M and SRA identifiers of all metagenomes and metatranscriptomes, along with detailed information for each sample, are available in Supplementary Tables 1 and 2, with extended physicochemical parameters available on ESS-DIVE (https://ess-dive.lbl.gov). Specific commands and options used for read filtering are available for each library on the JGI Data portal (https://data.jgi.doe.gov), under proposal ID 503568 (https://genome.jgi.doe.gov/portal/Thesynhydregimes/Thesynhydregimes.info.html).

## Abbreviations

dsDNA: Double-stranded DNA
ssDNA: Single-stranded DNA
ssRNA: Single-stranded RNA
dsRNA: Double-stranded RNA
KTW: Kill-the-Winner
PTW: Piggyback-the-Winner
ERW: East River Watershed
KO: KEGG Orthology
EC: Enzyme commission
UViGs: Uncultivated virus genomes
vOTU: Viral operational taxonomic unit
RPKM: Reads per kilobase per million
RdRP: RNA-dependent RNA polymerase
RT: Reverse transcriptases
AAI: Amino acid identity
PTL: Piggyback-The-Loser
PTP: Piggyback-The-Persistent
