# Supplementary material for: Virus diversity and activity is driven by snowmelt and host dynamics in a high-altitude watershed soil ecosystem
Source: Microbiome. 2023 Oct 27;11:237. doi: 10.1186/s40168-023-01666-z (PMC10604447; doi:10.1186/s40168-023-01666-z)
Supplement: Supplementary file 2 — Additional file 1: Supplementary Fig. 1. Overview of the Pipeline for ERW Soil Sample Analysis. Supplementary Fig. 2. Distribution of coverage breadth and coverage depth in metatranscriptomes for DNA and RNA vOTUs. Supplementary Fig. 3. Diversity and phylogenetic analyses of ERW RNA viral communities. Supplementary Fig. 4. Completeness, global distribution, virus taxonomy, and host taxonomy for all DNA vOTUs (< 10 kb + ≥ 10 kb). Supplementary Fig. 5. Temporal dynamics of total and active DNA and RNA viral communities. Supplementary Fig. 6. Activity of DNA phages. Supplementary Fig. 7. Temporal dynamics of non-significant virus-host relationships. [file 40168_2023_1666_MOESM1_ESM.docx]

**ADDITIONAL FILE 1**

**Supplementary Figure 1. Overview of the Pipeline for ERW Soil Sample Analysis. A.** vOTU set generation. The first part of the figure illustrates the initial steps of the pipeline, starting from the collection of soil samples (n = 48) and progressing towards the generation of a vOTU set. This process involves sample processing, DNA and RNA extractions, sequencing to generate metagenome and metatranscriptome assemblies, as well as a combined assembly, viral contig prediction, curation, and clustering. All steps are used to generate an OTU set. **B.** Downstream Analyses of the vOTU set. The second part of the figure focuses on the downstream analyses performed using the vOTU set generated in the previous step. These analyses encompass taxonomic classification, functional annotation, read mapping, host prediction, and activity analyses, which collectively provide insights into the viral composition, functional potential, host interactions, and activity levels in the ERW soil samples.

**
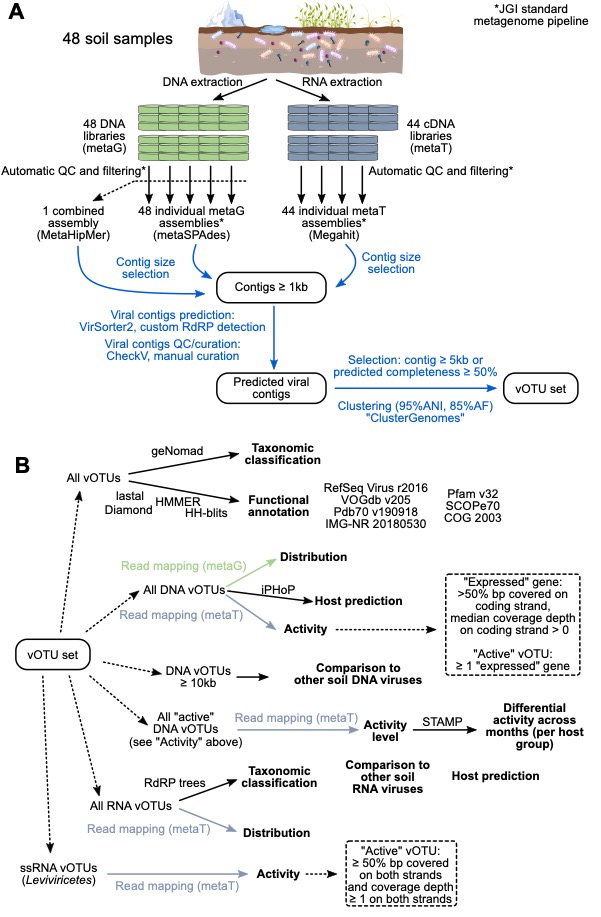
**

**Supplementary Figure 2. Distribution of coverage breadth and coverage depth in metatranscriptomes for DNA and RNA vOTUs.** The top panels show the distribution of coverage breadth (top right), i.e., number of bp covered by reads, and median coverage depth (top left) for the coding strand of genes from individual vOTUs and individual metatranscriptomes. A dashed outline shows the category of genes considered as "expressed", i.e., with 50% of bases covered or more and a median coverage depth of 1 or more. The middle panels show the distribution of the number of genes considered as expressed by DNA vOTU, for all vOTUs (left) or only vOTUs longer than 10kb (right). The bottom panel shows the coverage breadth for non-coding strand of +ssRNA viruses, i.e., the percentage of bases covered for the negative strand generated during active replication. The dashed outline shows the category of +ssRNA viruses considered as "actively replicating", i.e., 50% or more of bases covered on the negative strand.

**
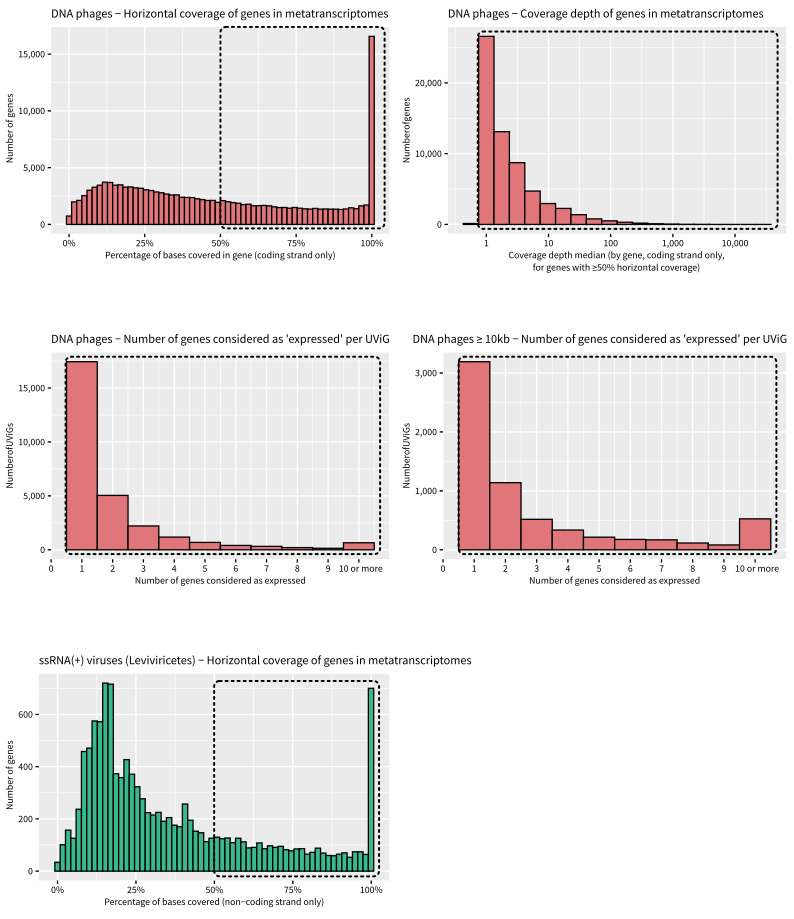
**

**Supplementary Figure 3.** **Diversity and phylogenetic analyses of ERW RNA viral communities**. Rooted phylogenetic trees of RdRP sequences belonging to the *Pisuviricota* (Supplementary Figure 3A), *Kitrinoviricota* (Supplementary Figure 3B), *Duplornaviricota* (Supplementary Figure 3C), and *Negarnaviricota* (Supplementary Figure 3D) phyla. The trees are rooted using reverse transcriptases as an outgroup and visualized with *ggtree.* Clusters composed exclusively of ERW sequences are colored in brown (ring 1) with branches leading to these clusters highlighted in light brown in the tree, while clusters composed of ERW sequences and existing virus sequences are colored by the environment type of the study (soil: dark brown, aquatic: blue, public databases: dark grey). Virus taxonomy (ring 3) and host (ring 4) are predicted based on the position of reference sequences from the RefSeq database in the tree (see Methods). Relative abundance of RPKM of *Pisuviricota* (Supplementary Figure 3E), *Kitrinoviricota* (Supplementary Figure 3F), *Duplornaviricota* (Supplementary Figure 3G), and *Negarnaviricota* (Supplementary Figure 3H) phyla.

| 1. ***Pisuviricota*** | **E** |
| --- | --- |
| **** | **** |
| 1. ***Kitrinoviricota*** | **F** |
| **** | **** |
| 1. ***Duplornaviricota*** | **G** |
| **** | **** |
| 1. ***Negarnaviricota*** | **H** |
| **** | **** |

**Supplementary Figure 4.** **Completeness, global distribution, virus taxonomy, and host taxonomy for all DNA vOTUs (<10kb + ≥10kb).** Global distribution is based on shared clusters from a vContact2 analysis, virus and host taxonomy are based on GeNomad and iPHoP tools, respectively (see Methods).

**Supplementary Figure 5. Temporal dynamics of total and active DNA and RNA viral communities.** Upset plots of the distribution of DNA (Supplementary Figure 5A) and RNA (Supplementary Figure 5B) vOTUs by month, in which vOTUs are grouped based on the (combination of) month(s) they were detected in. Boxplots (Supplementary Figure 5C) of Bray-Curtis dissimilarities between months and within months for DNA and RNA phages, and eukaryotes-infecting RNA viruses.

**Supplementary Figure 6. Activity of DNA phages.** Proportion of active (dark and light red), inactive (light grey), and absent (dark grey) DNA phages across months for predicted temperate (Supplementary Figure 6A) and predicted lytic (Supplementary Figure 6B) DNA vOTUs using BACPHLIP. Within DNA vOTUs identified as active, the ones likely engaged in active lytic infection was identified based on the functional annotation of expressed genes, while other active vOTUs are identified as “Active - Unknown”. A vOTU is considered as active in a given month when it is detected as active in at least one sample. The proportion of active vOTUs for each month is the sum of all active vOTUs for a given month.

**A**

**B**

**Supplementary Figure 7. Temporal dynamics of non-significant virus-host relationships. A.** Temporal dynamics of active DNA phages that didn’t exhibit a significant of changes in active DNA vOTU abundance between months (n = 2,749). The significance of changes in abundance between months was tested with a multiple group statistic test (ANOVA), a post-hoc test (Tukey-Kramer) to identify which pairs of months differ from each other and a multiple test correction (Storey’s FDR) to control false discovery rate, using STAMP. The seasonal dynamics of each non-significant active DNA vOTUs was plotted using the mean of metatranscriptomic RPKM transformed in z-score. Finally, vOTUs’ dynamics are grouped by panel, depending of the “ecology strategy” of their assigned host (see Methods). Each host were associated to an “ecological strategy” depending to the month (or season) a given host was supposed to be growing[6], represented by colored boxes in each panel. Finally, all active DNA vOTUs without assigned host or host without a clear ecological strategy were plotted in the last panel. **B.** Boxplot of standard-error values (log scale) for non-Significant (dark grey) and significant (light grey) active DNA vOTUs by month. The significance levels are determined through Analysis of Variance (ANOVA) tests, comparing differences among the standard-error values between non-significant and significant active DNA vOTUs’ pairs, at each month.
